# Protocol Adherence in Prehospital Medical Care Provided for Patients with Chest Pain and Loss of Consciousness; a Brief Report 

**Published:** 2017-01-11

**Authors:** Mostafa Mehrara, Nader Tavakoli, Marzieh Fathi, Babak Mahshidfar, Mohammad Amin Zare, Azita Asadi, Saeedeh Hosseinzadeh, Mehdi Safdarian

**Affiliations:** 1 Department of Emergency Medicine, Hazrat-e-Rasoul Akram Medical Centre, Iran University of Medical Sciences, Tehran, Iran.

**Keywords:** Emergency medical services, protocol adherence, chest pain, unconsciousness, medical audit

## Abstract

**Introduction:**

Although many protocols are available in the field of the prehospital medical care (PMC), there is still a notable gap between protocol based directions and applied clinical practice. This study measures the rate of protocol adherence in PMC provided for patients with chest pain and loss of consciousness (LOC).

**Method:**

In this cross-sectional study, 10 educated research assistants audited the situation of provided PMC for non-traumatic chest pain and LOC patients, presenting to the emergency department of a tertiary level teaching hospital, compare to national recommendations in these regards.

**Results:**

101 cases with the mean age of 56.7 ± 12.3 years (30-78) were audited (55.4% male). 61 (60.3%) patients had chest pain and 40 (39.7%) cases had LOC. Protocol adherence rates for cardiac monitoring (62.3%), O2 therapy (32.8%), nitroglycerin administration (60.7%), and aspirin administration (52.5%) in prehospital care of patients with chest pain were fair to poor. Protocol adherence rates for correct patient positioning (25%), O2 therapy (75%), cardiac monitoring (25%), pupils examination (25%), bedside glucometery (50%), and assessing for naloxone administration (55%) in prehospital care of patients with LOC were fair to poor.

**Conclusion:**

There were more than 20% protocol violation regarding prehospital care of chest pain patients regarding cardiac monitoring, O2 therapy, and nitroglycerin and aspirin administration. There were same situation regarding O2 therapy, positioning, cardiac monitoring, pupils examination, bedside glucometery, and assessing for naloxone administration of LOC patients in prehospital setting.

## Introduction

Prehospital medical care (PMC) is a vital component of medical care system which affects the outcome of whole health system by saving the patient’s golden time-to-treatment and changing his prognosis especially in patients with potentially life-threatening complaints like chest pain (1-3). 

Because of the importance of providing on-time high-quality PMC, several national and international evidence-based guidelines are prepared for emergency medical services (EMS) staff which advocate a particular course of action in clinical care and help to choose and perform the most effective interventions as prompt as possible (4). While pre-determined consensus instructions are available widely, there is still a notable gap between protocol based directions and applied clinical practice of EMS staff (5-7). This gap may result in incomplete or inappropriate care providing and increased morbidity and mortality. Thus measuring the rate of protocol adherence is a useful tool for evaluating the quality and comprehensiveness of medical care delivered in prehospital settings and probing the protocols to find the potential defects and insufficiencies which may lead to protocol violation by care providers. 

There are different studies evaluating the rate of protocol adherence in prehospital and hospital environments and patients with different clinical situations (like cardiac arrest, traumatic brain injury, sepsis, myocardial infarction, seizure, asthma and etc.) (7-12). In our knowledge this is the first study in our country which measures the rate of protocol adherence in prehospital care provided for patients with chief complaints of chest pain and loss of consciousness in an urban public referral system. 

## Methods

***Study design and setting***

This cross-sectional single-center study was conducted in a tertiary level teaching hospital with total annual census of 40,000 adult patients in May-June 2013. Study was approved by the institutional ethics committee (faculty of medicine, Iran University of Medical Sciences) and carried out in accordance with Declaration of Helsinki (1989). Informed written consent was obtained from patients or their legal guardians to use their data.

***Participants***

We enrolled conveniently the ≥18 year old patients with chief complaints of non-traumatic chest pain and loss of consciousness who was transferred to hospital by public EMS and admitted in our emergency department. Patients who had taken any medical care in other facilities before transferring to our hospital and patients who had used any medications before the arrival of EMS were excluded from study. Patients who had experienced cardiac arrest and needed the use of prehospital cardiac arrest protocol and patients with specific known or highly probable causes of loss of consciousness (like head trauma and intoxications) were excluded from study.

***Procedure***

For evaluating the protocol adherence 10 research assistants who were medical students were educated and informed about the national protocols for essential medical care which should be delivered to patients in prehospital setting in a 4-hour didactic course. In this course they became familiar with a checklist consisted of questions about the basic elements of protocol(s) which should be used by EMS care providers in approaching the patients with chest pain and loss of consciousness. Then they completed the checklist for 15 EMS transported patients under supervision of an emergency physician. These 15 patients were not included in study. After completing the preliminary checklists, these research assistants attended in different clinical day and night shifts in emergency department and evaluated the protocol adherence in provided prehospital care by checking the items considered in checklists. In challenging cases or items, they consulted with on-duty emergency physician. 

In patients with chief complaint of non-traumatic chest pain the most important items of our national protocol which should be evaluated were: measuring and documenting the blood pressure, beginning the cardiac monitoring and oxygen-therapy, maintaining a peripheral intra-venous line, administrating nitroglycerine and aspirin in cases suspicious to acute coronary syndrome, assessing the probability of pneumothorax (by auscultation the lung sounds)(13). 

In patients with chief complaint of loss of consciousness the most important items of our national protocol which should be evaluated were: assessing the airway and the patient’s need to airway management and performed interventions in this domain including the use of manual maneuvers to orotracheal intubation, beginning the cardiac monitoring and supplemental oxygen, putting the patient in anti-aspiration position, performing bedside glucometery, examining the pupil size, administrating the naloxone and 50% dextrose (if indicated) (13).

***Data Analysis***

Minimum needed sample size was calculated as 101 patients according to “*n=t*^2^pq / (p*δ)^2^” formula. All data analyses were performed with SPSS version 18 (SPSS, Inc., Chicago, IL, USA). Continuous numerical data were reported as mean ± standard deviation and categorical variables were presented with frequency and percentage. The adherence rates were categorized into five groups based on Likert scale: ≥ 90% as excellent, 80-90% good, 70-80% fair, 60-70% weak and < 60% poor. 

## Results

PMC of 137 patients were studied (80 patients with non-traumatic chest pain and 57 patients with loss of consciousness). 36 cases were exclude because of following criteria: 2 patients had experienced prehospital arrest and resuscitated by EMS staff, 16 patients had received medical care in another facility before calling EMS, 11 patients had used sublingual nitroglycerine before calling EMS, 3 cases with loss of consciousness were very suspicious to opium overdose, 1 case was suspicious to have tri-cyclic antidepressant overdose (depending on their history), and 3 cases had suspicious history of head trauma. Finally 101 cases were enrolled for analysis. 

***Baseline characteristics***

PMC of 101 cases with the mean age of 56.7 ± 12.3 years (30-78) were audited (55.4% male). 61 (60.3%) patients had chest pain and 40 (39.7%) cases had LOC. 35 (34.6%) of patients had previous history of ischemic heart disease, 27 (26.7%) diabetes mellitus, 30 (29.7%) hypertension, 12 (11.8%) hyperlipidemia, 6 (5.9%) drug/substance abuse. 

***Protocol adherence***

Table 1 and 2 summarize the results of analysis regarding the protocol adherence rates of provided PMC for patients with chest pain and LOC. Protocol adherence rates for cardiac monitoring (62.3%), O2 therapy (32.8%), nitroglycerin administration (60.7%), and aspirin administration (52.5%) in prehospital care of patients with chest pain were fair to poor. Protocol adherence rates for correct patient positioning (25%), O2 therapy (75%), cardiac monitoring (25%), pupils examination (25%), bedside glucometery (50%), and assessing for naloxone administration (55%) in prehospital care of patients with LOC were fair to poor. 

**Table 1:** Protocol adherence rate of provided prehospital medical care for patients with chest pain (n=61)

**Protocol based instruction **	**Adherence rate**
**Assessing the probability of pneumothorax **	61 (100.0)
**Beginning the cardiac monitoring **	38 (62.3)
**Measurement of vital signs **	58 (95.1)
**Beginning the oxygen therapy **	20 (32.8)
**Peripheral intravenous line **	59 (96.7)
Line is open and working	54 (91.5)
Line does not work	5 (8.4)
**Administration of nitroglycerine **	37 (60.7)
**Administration of aspirin **	32 (52.5)

Data were presented as frequency and percentage.

**Table 2:** Protocol adherence rate of provided prehospital medical care for patients with loss of consciousness (n=40

**Protocol based instruction **	**Adherence rate**
**Airway management**	36 (90.0)
**Correct patient positioning **	10 (25.0)
**Beginning the oxygen therapy **	30 (75.0)
**Beginning the cardiac monitoring **	10 (25.0)
**Measurement of vital signs **	40 (100.0)
**Examining the pupils **	10 (25.0)
**Peripheral intravenous line **	40 (100.0)
Line is open and working	38 (95.0)
Line does not work	2 (5.0)
**Bedside glucometery **	20 (50.0)
**Assessing the indication of naloxone **	22 (55.0)

Data were presented as frequency and percentage.

## Discussion

Based on the results of present study, protocol adherence regarding cardiac monitoring, O2 therapy, and nitroglycerin and aspirin administration in prehospital care of chest pain patients were fair to poor. Regarding pre hospital care of LOC patients, there were fair to poor protocol adherence for patient positioning, O2 therapy, cardiac monitoring, pupils examination, bedside glucometery, and assessing for naloxone administration. Except some limited items like assessing the probability of pneumothorax and airway management, other protocol based instructions are not followed by EMS staff. Even vital signs measurements are not done completely and available simply-provided cares like oxygen therapy are not provided satisfactorily. Our study showed also that the interventions both in monitoring and treatment sectors are under-mentioned by EMS staff and beginning cardiac monitoring is as neglected as administration of nitroglycerine and aspirin in patients with chest pain. According to our results, patients in loss of consciousness group received less medical care in prehospital setting than patients with chest pain. This is while the approach to patients with both complaints are illustrated in available step-by-step well established protocols which are taught tremendously in almost all clinical courses implemented for EMS staffs. 

Our findings about oxygen therapy is compatible with the findings of Hale et al who showed in their study that although oxygen use in ambulances is a very common daily procedure, protocol adherence in its use in suboptimal especially in some patients like patients with chronic obstructive pulmonary diseases (14). 

The protocol violation has been issued in other studies too. For example a retrospective study on paramedic compliance with advanced cardiac life support (ACLS) epinephrine guidelines in 75 out-of-hospital cardiac arrest showed that only 14% of patients received epinephrine in accordance with current ACLS guidelines (14). Another study by Johansson et al showed that in the majority of out-of-hospital cardiac arrest cases epinephrine is not administered according to current ACLS guidelines and the adherence to guidelines is lower in out-of-hospital cardiac arrest than in in-hospital ones (14). Similar studies on quality of out-of-hospital resuscitation showed similar results. As the Wik et al showed in their study on out-of-hospital resuscitation that chest compressions were not delivered half of the time, most of delivered compressions were too shallow and patients’ golden time was wasted without trying to do the chest compressions, electrocardiographic analysis, defibrillation or any other interventions (6). This is while most studies on this era have shown that protocol adherence can improve the management and clinical outcomes of patients (15-17). 

Protocol violation is not a problem only in prehospital setting and it is also seen in emergency department and other inpatients settings (18). Some efforts (like presence of a pharmacist on the resuscitation team, simplification of protocols, changing the protocols, etc.) have been done to improve the compliance with guidelines but none of them are approved in this era (19). 

For better conclusion in this regard performing a complete KAP (knowledge, attitude, practice) study with larger sample size could be helpful.

Therefore, more attention in this field and theoretical and practical training courses in this field seems to be helpful.

## Conclusion:

There were more than 20% protocol violation regarding prehospital care of chest pain patients regarding cardiac monitoring, O2 therapy, and nitroglycerin and aspirin administration. There were same situation regarding O2 therapy, positioning, cardiac monitoring, pupils examination, bedside glucometery, and assessing for naloxone administration of LOC patients in prehospital setting.
